# Metal Concentrations in Soil Paste Extracts as Affected by Extraction Ratio

**DOI:** 10.1100/tsw.2002.169

**Published:** 2002-04-09

**Authors:** Filip M.G. Tack, Nic Dezillie, Marc G. Verloo

**Affiliations:** Laboratory of Analytical Chemistry and Applied Ecochemistry, Ghent University, Coupure Links 653, B-9000 Gent, Belgium

**Keywords:** heavy metals, soils, soil solution, mobility, saturated paste extract

## Abstract

Saturated paste extracts are sometimes used to estimate metal levels in the soil solution. To assess the significance of heavy-metal concentrations measured in saturation extracts, soil paste extracts were prepared with distilled water in amounts ranging from 60–200% of the moisture content at saturation. Trace metals behaved as if a small pool consistently was dissolved independent of the extraction ratio applied. Metal concentrations in the solution hence were not buffered by the solid phase, but the observed behaviour would allow the estimation of metal concentrations in the soil solution as a function of moisture content. The behaviour of iron and manganese suggested that some microbial reduction occurred. The intensity increased with increasing extraction ratio but not to the extent of affecting dissolution of trace elements.

## INTRODUCTION

Potential adverse effects of heavy metals are most likely determined by their contents in the soil solution. With respect to plant uptake, it is assumed that the activity of the free metal ion in solution largely governs metal contents in crops[1,2]. In contaminated soils, leaching phenomena and the related risk of groundwater contamination are directly proportional to the concentrations in the soil solution, which in turn are determined by the physicochemical soil environment[3,4].

Although highly desirable in environmental research, sampling the true soil solution without altering its chemistry is faced with difficulties. Saturated paste extracts may provide a more practical alternative to obtain information about the soil solution. A saturated paste extraction of a soil is a method originally developed to determine salts in soils[5], but workers in heavy-metal research also have relied on it to obtain a reflection of metal levels in the soil solution[4,6,7,8,9,10,11,12].

**TABLE 1**
**Characteristics of the Investigated Soil**

| | |
|---|---|
| **Soil Properties** | |
| pH | 8.1 |
| Organic matter | 1.1% |
| $CaCO_3$ | 4.9% |
| 0–2 µm | 9.4% |
| 2–50 µm | 16.4% |
| 50 µm to 2 mm | 74.2% |
| **Trace Metal Contents (mg $kg^{-1}$ DM)** | |
| Cd | 0.6 |
| Cu | 25.8 |
| Pb | 49.1 |
| Zn | 196 |
| Mn | 490 |

The aim of this work was to investigate how heavy-metal concentrations in an aqueous extract are affected by the extraction ratio. This may provide more insight into the factors controlling metal concentrations in the soil solution and, from a practical point of view, may enhance our understanding of the significance of heavy-metal concentrations measured in saturation extracts.

## MATERIALS AND METHODS

A surface soil was sampled in August 2000 from a confined, dredged-sediment disposal site in Menen, Belgium[13]. Disposal of dredged sediments ceased in 1994. The sampled location was fallow land covered with herbs and grasses. Soil was sampled from 20–40 cm using an auger corer, air dried, and passed through a 2-mm sieve. Selected properties and elemental contents of the soil are presented in Table 1.

The soil was subjected to extractions with deionised water. The basic procedure was the extraction of a saturated paste as described by Jackson[5], with variation in the amounts of water added for extraction. These amounts corresponded to 60, 80, 90, 100, 120, and 200% of the saturation point of the soil (255 ml $kg^{-1}$ dry soil). The soil was allowed to equilibrate during 12 h. The solution was obtained by vacuum filtration on a Büchner filter fitted with filter paper. A portion of the obtained liquid was filtered through an acid-rinsed 0.45-µm membrane filter and acidified to pH 2 using concentrated ultrapure $HNO_3$ for analysis of the metals. The remaining extract was conserved at 3ºC for at most 12 h before analysis of the anions with ion chromatography (Dionex Corp., Sunnyvale, CA).

Soil metal contents are pseudototal contents as determined by aqua-regia digestion[14]. Metals were determined by flame atomic absorption (Varian SpectrAA-10, Palo Alto, CA) equipped with deuterium correction or with graphite furnace equipped with Zeeman background correction (Varian SpectrAA-100, Palo Alto, CA). Quality was controlled by the analysis of duplicates, procedural blanks, and standard reference materials (estuarine sediment CRM 277 and light sandy soil CRM 142 R). Soil pH was determined in the saturation extract. Calcium carbonate was determined by acid-base titrimetry[15]. Organic carbon was measured using the method of Walkley and Black and expressed as organic matter using the factor 1.72[16]. Particle-size analysis was performed by conventional dry and wet seeving after destruction of organic matter and carbonates[17].

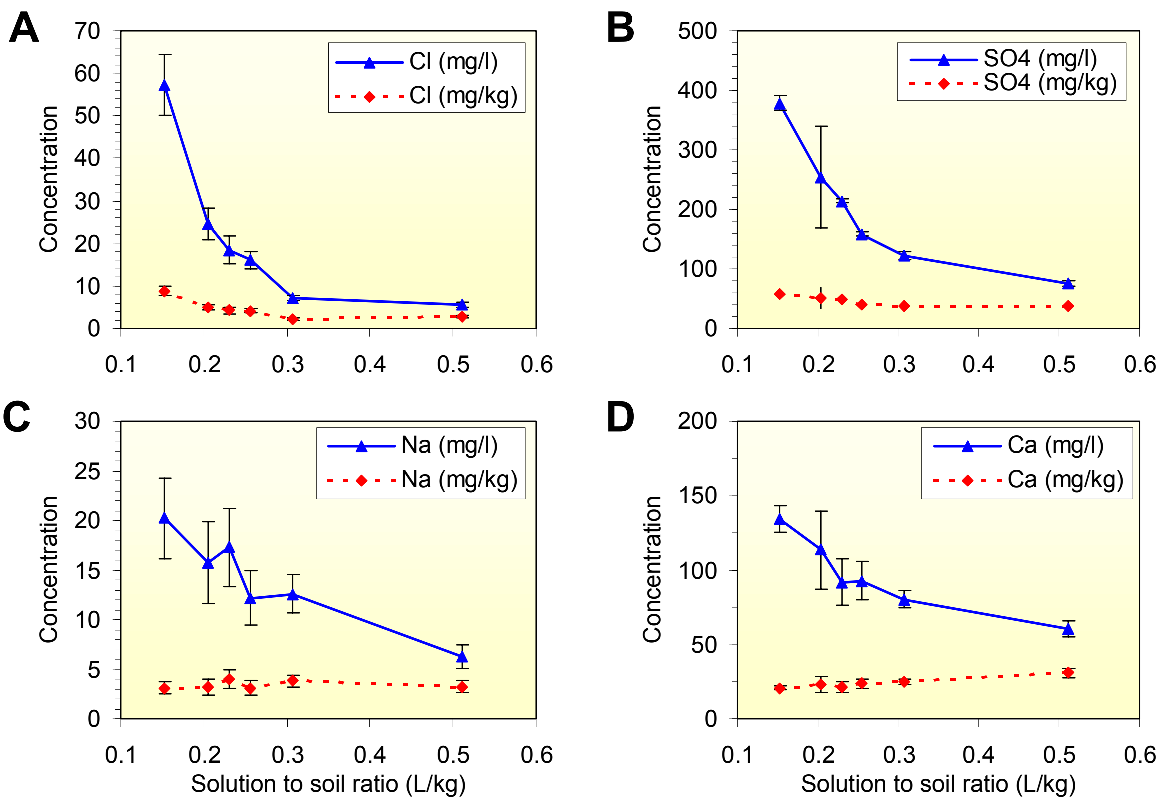

**FIGURE 1**. Concentrations in aqueous extracts (solid lines) and extracted amounts (dotted lines) of $Cl^-$, $SO_4^{2-}$, Na, and Ca as a function of extraction ratio. Error bars denote the standard deviation ($n = 3$).

## RESULTS AND DISCUSSION

The soil was characterised as a sandy loam according to the USDA texture classification[18]. The soil pH was rather high at 8.1. This is consistent with the presence of free carbonates. Contents of copper were within normal ranges for soils with a similar clay and organic carbon content in Flanders, Belgium (18–30 mg $kg^{-1}$)[19]. The other elements were slightly elevated, about two times higher than normal baseline concentration levels for cadmium and lead (0.1–0.4 and 18–28 mg $kg^{-1}$, respectively) and about four times higher for zinc (32–45 mg $kg^{-1}$) (Table 1).

The experimental data show that for most elements, concentration decreased hyperbolically with increasing amount of water used for extraction (Figs. 1 to 3). When expressed on the basis of dry soil, extracted quantities of the elements remained constant. The system therefore behaved largely as if a water-soluble pool of elements consistently is released into solution independent of the moisture content during extraction.

This behaviour implies that the solid phase is not buffering metal concentrations in the solution. Soil solution metal concentrations in the field could therefore be expected to vary significantly with the moisture content for this soil. This will be an important factor in determining the actual mobility and availability of metals in the field and may need more consideration to improve the quality of risk assessment. Clearly, concentrations measured in the saturated paste extract will be different from concentrations in the actual soil solution. The observed behaviour, however, allows the easy estimation of metal concentrations in the soil solution as a function of soil moisture content from a single determination of metal concentrations in the saturation extract. There is a great need to investigate other soils in a similar way.

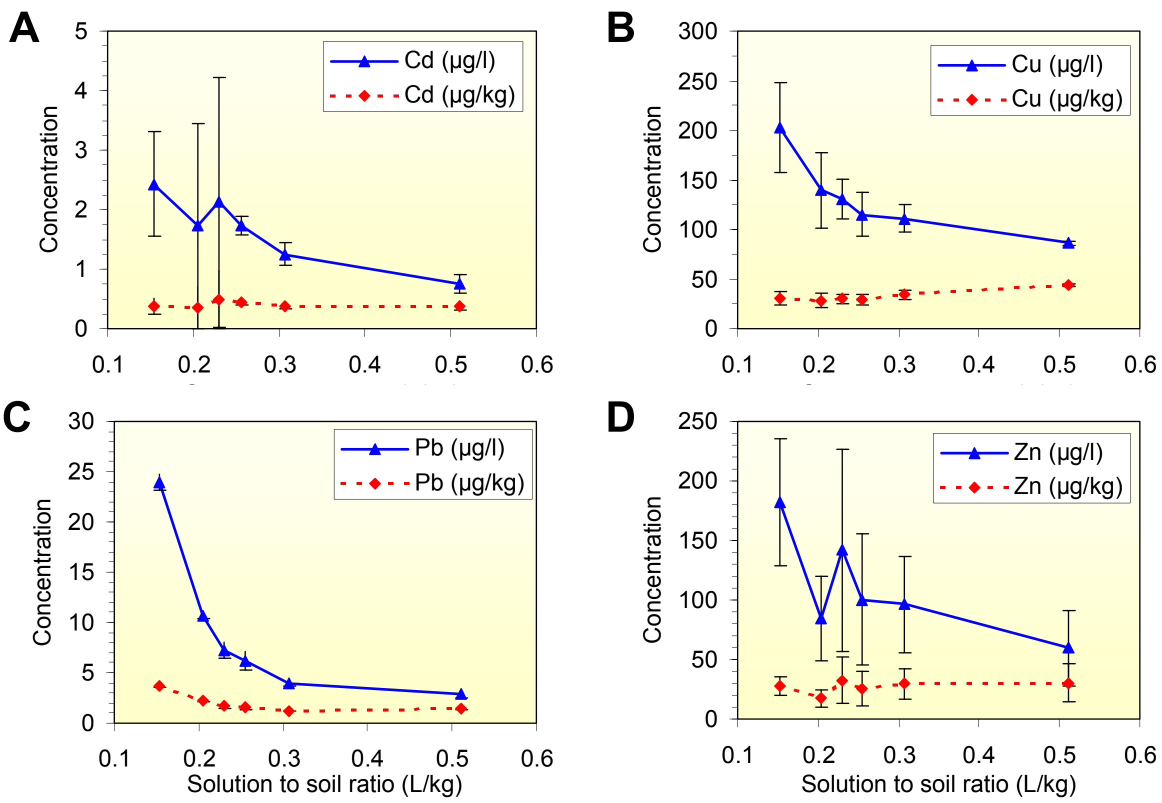

**FIGURE 2**. Concentrations in aqueous extracts (solid lines) and extracted amounts (dotted lines) of Cd, Cu, Pb, and Zn as a function of extraction ratio. Error bars denote the standard deviation ($n$ = 3).

Calcium concentrations decreased with increasing extraction ratio (L/S ratio); but expressed per kilogram of soil, amounts extracted increased slightly rather than remaining constant (Fig. 1D). This behaviour is not consistent with the observation that the soil contained 5% of free carbonates. From thermodynamics, it would be expected that with increasing extraction ratio, increasing amounts of $CaCO_3$ would go into solution to meet the solubility product of soil $CaCO_3$[20]. The system in true thermodynamic equilibrium should therefore exhibit rather constant calcium concentrations with increasing L/S ratio. The currently observed behaviour reflects kinetic constraints. At pH = 7.2, or a proton concentration of $10^{-7.2}$ mol $l^{-1}$, dissolution of calcium carbonates may indeed proceed very slowly.

For iron and manganese, extracted amounts increased strongly with extraction ratio (Fig. 3). This can be accounted for by microbial reduction processes. It is observed from the graph that the increase was pronounced for extraction ratios above the saturation point of the soil. Saturated and oversaturated moisture conditions will favour the occurrence of anoxic zones and result in an increasingly important reduction of iron and manganese after 12 h of equilibration. Iron manganese oxides play a very important role in metal retention in soils[21], and reduction of these phases therefore may initially result in a release of metals. In the current experiment, extracted iron and manganese remained very limited compared to total amounts, which may be the reason that there was not yet a significant effect on the trace metals. Even though locally reducing zones may occur after 12 h, the saturated soil paste remained essentially oxic. Tack et al.[4] observed that strongly reducing conditions were being established after about five consecutive saturated paste extractions.

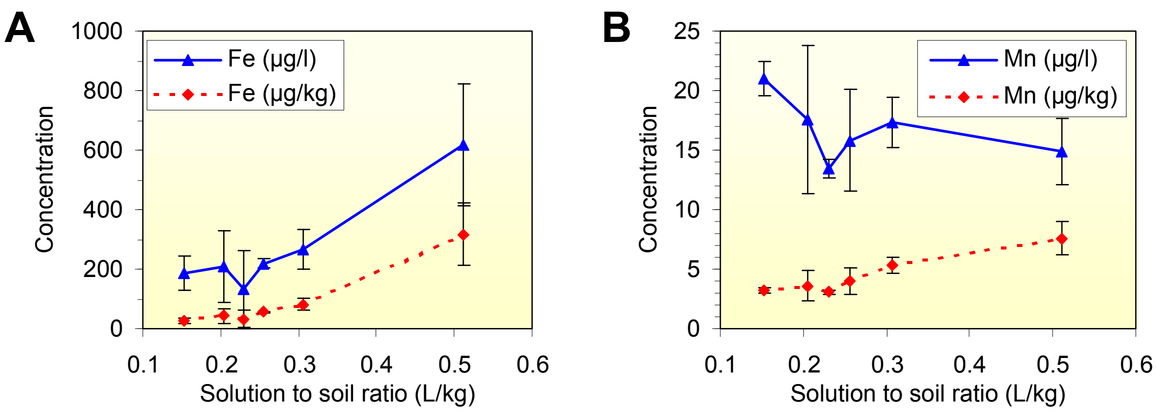

**FIGURE 3**. Concentrations in aqueous extracts (solid lines) and extracted amounts (dotted lines) of Fe and Mn as a function of extraction ratio. Error bars denote the standard deviation ($n = 3$).

The current experiment allowed a better appreciation of the practical significance of metal concentrations in a saturated paste extract. It is important to note that concentrations in the soil solution may not be effectively buffered by the soil solution. This may lead to significant variations in metal concentrations in the soil solution, which may have implications on environmental risk assessment, as it will affect the extent of leaching or plant uptake. Conclusions may not be generalised, however, before more soils have been investigated in a similar way.
